# Regional variation in myocardial blood flow in patients with left bundle branch block evaluated with quantitative perfusion CMR

**DOI:** 10.1186/1532-429X-15-S1-P205

**Published:** 2013-01-30

**Authors:** Manish Motwani, Laura Dobson, Craig D Smith, Neil Maredia, Steven Sourbron, John D Biglands, Sven Plein, John P Greenwood

**Affiliations:** 1 MCRC & LIGHT, University of Leeds, Leeds, UK; 2 Medical Physics, University of Leeds, Leeds, UK

## Background

This study is the first to use quantitative perfusion CMR to evaluate regional differences in myocardial blood flow (MBF) in patients with left bundle branch block (LBBB).LBBB is often associated with underlying CAD but its presence can limit the diagnostic accuracy of non-invasive imaging tests. In particular, there is a high incidence of false-positive results with exercise SPECT due to apparent septal perfusion defects. The use of vasodilator stress has reduced but not eliminated this problem. Several hypotheses have been postulated to explain the cause of such perfusion defects, and these include early activation of the septum, leading to shortened diastole and reduced blood flow; partial volume effects caused by impaired septal thickening; and increased septal intra-myocardial pressure during diastole, resulting in reduced flow reserve. A number of small studies using PET or early quantitative SPECT techniques have evaluated regional differences in MBF in patients with LBBB, but the results have been conflicting and have shown either no regional differences or a relative but not absolute reduction in septal perfusion. This study re-evaluates the unresolved question of septal perfusion in LBBB using quantitative perfusion CMR.

## Methods

9 patients with LBBB and no significant CAD underwent adenosine stress/rest perfusion CMR at 1.5T and X-ray coronary angiography. Absence of CAD was defined as luminal stenosis <40% on quantitative coronary angiography in all major vessels. Mid-ventricular perfusion data were segmented into 3 regions for each patient: septal, adjacent (anterior-inferior) and lateral. MBF and myocardial perfusion reserve (MPR) were then determined for the septal and lateral regions by Fermi function deconvolution.

## Results

**Figure 1:**
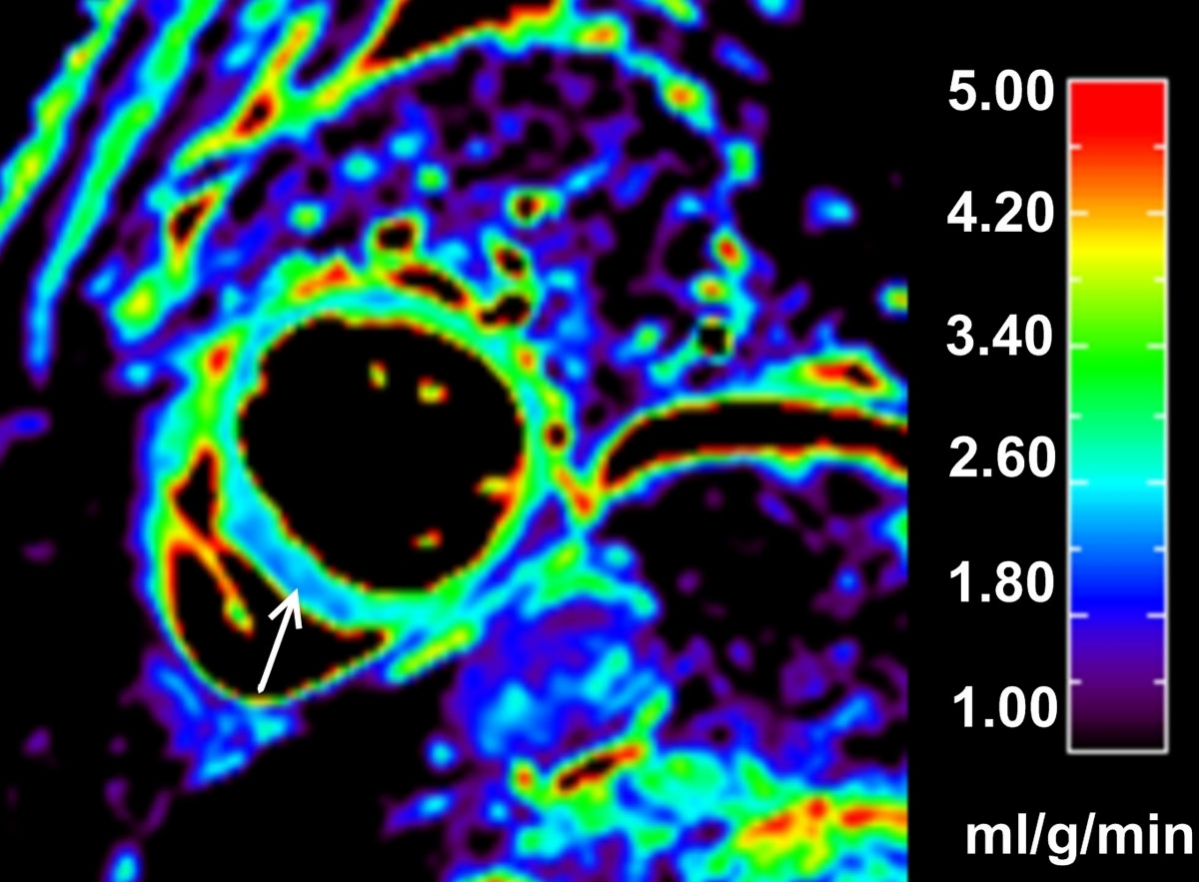
A 65 year old lady had LBBB on her ECG but normal coronary arteries on X-ray coronary angiography. A myocardial blood flow (MBF) map generated from quantitative perfusion CMR data at stress demonstrates a relative but not absolute reduction in septal perfusion (arrow, septal stress MBF = 2.76 ml/g/min) compared to the lateral region (lateral stress MBF= 3.74 ml/g/min).

Resting MBF was similar in both septal and lateral regions in all patients (1.27±0.26 vs. 1.27±0.23 ml/g/min; p=0.95) (Table 1). Stress MBF was significantly lower in septal regions compared to lateral regions in all patients (septal/lateral ratio = 0.86 ± 0.07) (Table 1, Figure 1). Accordingly, the mean stress MBF and mean MPR were significantly lower in the septal regions compared to lateral regions (MBF: 3.99±1.03 vs. 4.62±0.96 ml/g/min, p<0.001; MPR: 3.19±0.78 vs. 3.71±0.88ml/g/min, p<0.01). However, stress MBF and MPR estimates remained within the published normal range for both septal and lateral regions in all patients (Table 1).

**Table 1:** Regional Myocardial Blood Flow Estimates in Patients with LBBB

Patient	Stress MBF (ml/g/min)		Rest MBF (ml/g/min)		MPR	
	***Septal***	***Lateral***	***Septal***	***Lateral***	***Septal***	***Lateral***

**1**	4.73	4.83	1.42	1.46	3.33	3.31
**2**	2.56	3.33	0.91	1.11	2.81	3.00
**3**	3.60	4.45	1.40	1.44	2.57	3.09
**4**	5.17	5.76	1.23	1.08	4.20	5.33
**5**	4.71	5.53	1.42	1.48	3.32	3.74
**6**	4.07	4.52	1.52	1.17	2.68	3.86
**7**	3.11	3.53	0.80	0.84	3.89	4.20
**8**	5.24	5.88	1.29	1.32	4.06	4.45
**9**	2.76	3.74	1.48	1.54	1.86	2.43

***Mean***	3.99 ± 1.03	4.62 ± 0.96	1.27 ± 0.26	1.27 ± 0.23	3.19 ± 0.78	3.71 ± 0.88

***P value***	*p*<*0.001*		*p*=*0.95*		*p*<*0.01*	

MBF = myocardial blood flow MPR = myocardial perfusion reserve

## Conclusions

This study suggests that although septal perfusion remains normal in LBBB, there is a genuine relative reduction in MBF compared to the lateral wall. This phenomenon may account for the false positive results seen with myocardial perfusion imaging techniques and highlights a potential clinical utility of quantitative perfusion CMR.

## Funding

JPG and SP receive an educational research grant from Philips Healthcare. SP is funded by a BHF fellowship (FS/1062/28409).

